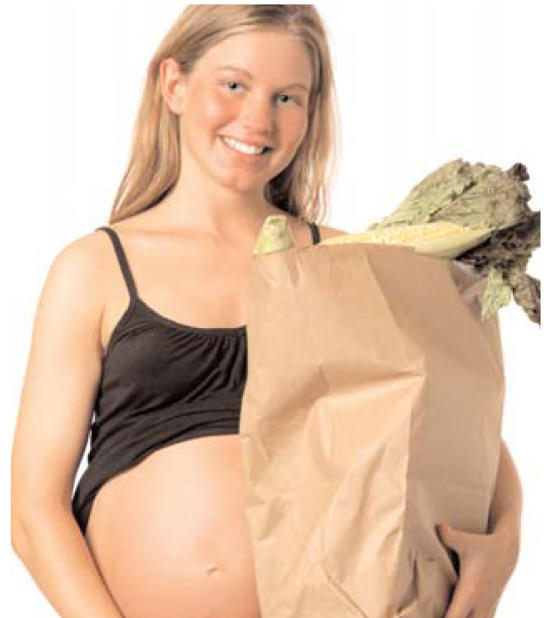# Diet and Nutrition: You Are What Your Mother Ate

**DOI:** 10.1289/ehp.115-a492b

**Published:** 2007-10

**Authors:** M. Nathaniel Mead

It is now axiomatic that the *in utero* environment influences prenatal development and may trigger structural and functional changes that can persist for a lifetime. New evidence of the importance of the womb environment for the long-term health of offspring was published in the June 2007 *Journal of Clinical Endocrinology and Metabolism*. The study’s findings show for the first time in humans that the diet a mother consumes in late pregnancy can alter the stress response of her offspring, possibly setting the stage for greater susceptibility to cardiovascular problems and other forms of stress-related disease into adulthood.

The study builds on an investigation published in the August 2003 issue of the same journal in which scientists from the United Kingdom found that greater maternal meat and fish intake were linked with elevated fasting plasma cortisol concentrations in offspring. The adrenal glands secrete cortisol in response to chronic stress, and this can affect both vascular responses and the metabolism of glucose and lipids. Epidemiologic studies have established that chronic elevation of cortisol levels in children and young adults can raise the risk of later developing hypertension, diabetes, and heart disease.

That original study population was a group of mothers attending a maternity hospital in Motherwell, Scotland, between 1952 and 1976. These mothers had been advised to eat a high-meat, low-carbohydrate diet in an experimental attempt to prevent preeclampsia (hypertension during pregnancy). The advice was to eat 1 pound of red meat daily during pregnancy and to abstain from carbohydrate-rich foods such as bread and potatoes. Subsequent studies showed that the adult offspring of mothers with the highest recorded meat intakes went on to experience high cortisol and develop hypertension.

For the most recent study, researchers recruited 31 men and 39 women born in Motherwell during 1967 and 1968. Previous analyses of the data abstracted from obstetric records had shown a doubling of protein intake between early and late pregnancy in the mothers of these children. The 70 adult offspring were given a 5-minute public speaking task followed by a 3-minute mental arithmetic task. Salivary cortisol was measured before, during, and after the tasks. Cortisol levels were found to increase in tandem with the amount of meat and fish the study participants’ mothers had consumed in late pregnancy. “Compared with offspring of mothers who had reported eating no more than 13 meat/fish portions per week, the average cortisol concentrations were raised by 22% and 46% . . . in offspring of those reporting 14–16 and at least 17 portions per week respectively,” the researchers wrote. The effect seemed slightly greater in men than women.

In animal studies, high-protein diets have been shown to stimulate the HPA axis, a component of the endocrine system that regulates cortisol and other stress-related hormones. “We are not certain of the mechanism, but postulate that an unbalanced diet in pregnancy may act as a stressor to the mother, increasing her production of cortisol and subsequently increasing cortisol production in the offspring,” says lead author Rebecca Reynolds, an endocrinologist at Queen’s Medical Research Institute in Edinburgh. In essence, by altering HPA axis activity and promoting high cortisol levels in the gestational environment, the high-protein diets appear to “reprogram” the developing fetal HPA axis.

Previous human studies related size at birth to adult plasma cortisol levels, but this is the first to examine the connection between gestational diet and stress adaptation in adult offspring. “This study adds to increasing evidence for the importance of the maternal diet,” says coauthor Keith Godfrey, an epidemiologist at the University of Southampton. Reynolds and her team now plan to examine how the increased stress responses in the Motherwell offspring relate to subsequent disease.

The Motherwell mothers adhered to a diet pattern similar to low-carb/high-protein diets that have recently become popular for weight control. Such diets have been associated with an increased risk of kidney problems and metabolic ketoacidosis, another potential prenatal stressor. Ketoacidosis results when carbohydrate intake is restricted and the body turns to other energy sources, such as fat; acids known as ketones build up in the blood. One of the by-products of ketoacidosis is beta-hydroxybutyrate. When this compound is elevated during gestation, it can stunt behavioral and intellectual development in offspring.

“In pregnant women, even mild ketoacidosis is a very serious condition that has an adverse effect on fetal growth and development,” says Natalia Igosheva, a research scientist at King’s College London. “Diet-induced metabolic ketoacidosis represents a possible mechanism for the impact of an unbalanced maternal diet on the offspring’s neurodevelopment.”

Instead of low-carb/high-protein diets, Reynolds urges women of child-bearing age to strive for a balanced diet. This is supported by a recent study out of Spain, published in the February 2007 issue of *Clinical Endocrinology*, in which women who chose a dietary pattern closer to the Mediterranean diet (which emphasizes fruits, vegetables, nuts, olive oil, chicken, and seafood) showed lower levels of HPA axis disturbance.

## Figures and Tables

**Figure:**